# Supplementary material for: Effect of Cytomegalovirus Reactivation on Inflammatory Status and Mortality of Older COVID-19 Patients
Source: Int J Mol Sci. 2023 Apr 6;24(7):6832. doi: 10.3390/ijms24076832 (PMC10094990; doi:10.3390/ijms24076832)
Supplement: Supplementary file 1 [file ijms-24-06832-s001.zip › ijms-2199252-supplementary.pdf]

# Effect of cytomegalovirus reactivation on inflammatory status and mortality of older COVID-19 patients

**Robertina Giacconi[1]*, Maurizio Cardelli [1], Francesco Piacenza[1], Elisa Pierpaoli[1], Elisabetta Farnocchia[1], Mirko Di Rosa[2], Anna Rita Bonfigli[3], Tiziana Casoli[4], Francesca Marchegiani[5], Fiorella Marcheselli[5], Rina Recchioni[5], Pierpaolo Stripoli[5], Roberta Galeazzi[6], Antonio Cherubini[7], Massimiliano Fedecostante[7], Riccardo Sarzani[8], Chiara Di Pentima[8], Piero Giordano[8], Roberto Antonicelli[9], Mauro Provinciali[1] and Fabrizia Lattanzio[3]**

[1] Advanced Technology Center for Aging Research, IRCCS INRCA, Ancona, Italy
[2] Unit of Geriatric Pharmacoepidemiology and Biostatistics, IRCCS INRCA, Ancona, Italy
3 Scientific Direction, IRCCS INRCA, Ancona, Italy
[4] Center for Neurobiology of Aging, IRCCS INRCA, Ancona, Italy
[5] Center of Clinical Pathology and Innovative Therapy, IRCCS INRCA, Ancona, Italy
[6] Clinical Laboratory and Molecular Diagnostic, Italian National Research Center on Aging, IRCCS INRCA, Ancona, Italy
[7] Geriatria, Accettazione geriatrica e Centro di ricerca per l'invecchiamento, IRCCS INRCA, Ancona, Italy
[8] Department of Clinical and Molecular Sciences, Università Politecnica delle Marche, Ancona, Italy; Internal Medicine and Geriatrics, Italian National Research Centre on Aging, Hospital "U. Sestilli", IRCCS INRCA, Ancona, Italy
[9] Cardiology Unit, IRCCS INRCA, 60129 Ancona, Italy.

* Correspondence: E-mail: r.giacconi@inrca.it; Advanced Technology Center for Aging Research, IRCCS INRCA, Via Birarelli 8, 60121 Ancona, Italy; Tel: +390718004213; Fax +39071206791

**Table S1 CMV IgM antibodies in COVID-19 patients**

| | CMV IgM n. (%) | | | |
|---|---|---|---|---|
| | **Negative** | **Positive** | **Not available** | **Dubious result** |
| **CMV DNAemia positive samples n. 19** | 11 (58%) | 4 (21%) | 2 (10.5%) | 2 (10.5%) |
| **CMV DNAemia negative samples n. 137** | 118 (86.1%) | 6 (4.4%) | 7 (5.1%) | 6(4.4%) |

**Table S2 Serum cytokine concentrations in COVID-19 patients according to CMV IgM or DNAemia positivity**

| | **CMV IgM negative** | **CMV IgM positive** | **CMV DNAemia positive** |
|---|---|---|---|
| IL-6 (pg/mL) | 157.6±24.9 | 133.3±87.1 | 149.9±74.1 |
| IL-10 (pg/mL) | 162.4±46.2 | 103.2±161.4 | 113.1±137.6 |
| IFN-Υ (pg/mL) | 53.4±11.9* | 57.0±31.5* | 137.6±30.9 |
| TNF-α (pg/mL) | 2.5±1.6** | 3.8±5.7* | 22.0±4.3 |

*p<0.05 compared to CMV DNAemia positive patients

**p<0.001 compared to CMV DNAemia positive patients
